# The Number of Remaining Teeth—A Predictability Factor for a Certain Type of Cardiovascular Condition in a Group of Hospitalized Individuals

**DOI:** 10.3390/jpm14121166

**Published:** 2024-12-21

**Authors:** Mirela Mihart, Veronica Mercuț, Sanda Mihaela Popescu, Monica Mihaela Iacov-Crăițoiu, Mihaela Ionescu, Adina Dorina Glodeanu, Alexandra Maria Rădoi, Monica Scrieciu

**Affiliations:** 1 Doctoral School, University of Medicine and Pharmacy of Craiova, 200349 Craiova, Romania; 2 Emergency Dentistry Department, County Emergency Clinical Hospital, 220044 Drobeta-Turnu Severin, Romania; 3 Department of Prosthodontics, University of Medicine and Pharmacy of Craiova, 200349 Craiova, Romania; veronica.mercut@umfcv.ro (V.M.); monica.iacov@umfcv.ro (M.M.I.-C.); monica.scrieciu@umfcv.ro (M.S.); 4 Department of Oral Rehabilitation, University of Medicine and Pharmacy of Craiova, 200349 Craiova, Romania; sanda.popescu@umfcv.ro; 5 Department of Medical Informatics and Biostatistics, University of Medicine and Pharmacy of Craiova, 200349 Craiova, Romania; 6 Department of Cardiology, University of Medicine and Pharmacy of Craiova, 200349 Craiova, Romania; adina.glodeanu@umfcv.ro

**Keywords:** number of remaining teeth, cardiovascular conditions, hospitalized subjects

## Abstract

**Background:** The aim of this study was to determine whether the oral parameter reflecting the total number of remaining permanent teeth (NRT) on both arches represents a predictability factor for a certain type of cardiovascular condition. **Methods**: This study included 84 subjects (40 males and 44 females) with ages between 50 and 89 years old, hospitalized in the Cardiology Department, who required dental examinations and specialized therapeutic interventions within the Emergency Dental Department of the same medical facility. **Results**: The study participant’s data were statically analyzed. An unadjusted oral parameter NRT < 21 may represent a statistically significant predictor of developing cardiomyopathy (OR = 8.00, 95%CI = 2.78–23.06, *p* < 0.0005), heart valve disease and arterial hypertension, in association with other comorbidities (except for metabolic or pulmonary comorbidities). The regression analyses revealed a borderline-significant association between the adjusted NRT and metabolic comorbidities or coronary disease (OR = 0.37, 95%CI = 0.13–1.01, *p* = 0.052). **Conclusions**: Overall, the NRT may be considered a predictive marker that is relative to the risk of exhibiting cardiovascular conditions.

## 1. Introduction

The oral cavity represents the first segment of the digestive system, playing a role in fulfilling certain functions such as mastication and phonation, and thus contributing to the homeostasis of the maxillofacial region [1]. Oral health represents a broad concept that is characterized by the presence of dental arches with a functional morphology. This allows individuals to continue with their desired social role, which is a fundamental aspect of general health, well-being and quality of life [2]. According to the World Health Organization (WHO), dental health constitutes one of the ten important criteria for the appreciation of human health [1].

Poor oral health was determined to be a potential risk factor for the appearance of cardiovascular events [3]. Numerous scientific proofs indicate that poor oral health is associated with the phenomenon of subclinical atherosclerosis and with cardiovascular diseases (CVD), although the existence of a causal relationship between these is still unclear [4,5]. Data from the specialty literature shows that the bond between oral conditions and CVD can be explained through the existence of the chronic inflammation phenomenon and the repeated bacteremia phenomenon, which take place in the oral cavity. It is well known that inflammation has an important role in the pathogenesis of atherosclerosis [6].

Investigation of the oral cavity can identify clinical signs indicating the presence of one systemic condition [7]. It is recommended that the oral examination is based on objective criteria, including counting the number of remaining natural teeth and a periodontal examination [8]. The number of teeth is a simple sign of oral health that is easy to understand and clinically applicable [9]. In the United States, during 2011–2016, 26% of adults aged 65 or older had less than eight remaining teeth, and about 17% of them had lost all their teeth [10]. Globally speaking, the prevalence of a severe loss of teeth (≤9 of remaining teeth) is about 2.4% [11].

The number of permanent natural teeth was evaluated in many epidemiological studies, which referred to an individual’s history of oral inflammatory conditions [12].

The main oral inflammatory conditions include dental caries, as well as apical and marginal periodontal diseases, with the most common ultimate consequence being tooth loss [13]. Because of that, the number of the remaining natural teeth is considered to be an indirect marker for the study of oral inflammatory conditions [3].

Holmlund et al. showed that the number of remaining natural teeth represents an adequate indicator for oral health, with a correlation between the number of the remaining natural teeth and CVD being presented for the first time [14].

Previous studies evaluated the association between the number of remaining natural teeth and stroke [15]. The possible differences in the association between oral diseases, the number of remaining natural teeth and CVD remain unclear [16]. Moreover, previous studies on this topic have been conducted on population samples from developed countries [17], limiting the extrapolation of the results to populations with different socio-economic and cultural characteristics. However, in the study conducted by Ogawa et al., it was indicated that the number of teeth is the most sensitive independent predictive marker of functional recovery after cardiovascular surgery [18]. Beukers et al. demonstrated that individuals with a lower number of remaining teeth consistently had a significantly higher risk of atherosclerotic cardiovascular events compared to individuals with a higher number of remaining teeth [19].

The main aim of the present study was to investigate whether the oral parameter reflecting the total number of remaining permanent teeth on both arches, hereinafter considered to be the number of remaining teeth (NRT), represents a predictability factor for a certain type of CVD, depending on certain variables, for the group of subjects studied. The influence of other systemic comorbidities on the association between NRT and a certain type of CVD was also investigated.

## 2. Materials and Methods

### 2.1. Study Design

This study was conducted on subjects hospitalized in Cardiology Department of the Emergency County Clinical Hospital Drobeta Turnu-Severin, who required dental examination and specialized therapeutic intervention within the Emergency Dental Service of the same hospital, between July and September 2022. The research included subjects hospitalized in the Cardiology Department, as studies in the field indicate that oral diseases are present in approximately 70% of hospitalized subjects [20]. It was also observed that the prevalence of oral diseases is higher among subjects with cardiovascular diseases in comparison to community-dwelling subjects without cardiovascular diseases [21]. The sample size was computed using G*Power 3.1.9.7, Heinrich Heine University Düsseldorf, Germany, considering a significance level α of 0.05, a power 1-β equal to 0.90 and a small effect size value of 0.15 (since there are not many data available in the literature, and with an awareness of practical significance), resulting in a study lot with a minimum of 73 participants.

This study was approved by the Ethics and Scientific Deontology Committee of the University of Medicine and Pharmacy Craiova, under approval no. 73/02.05.2022 and by the Ethics Council of the Emergency County Clinical Hospital Drobeta Turnu-Severin, under approval no. 06/14.07.2022, considering the criteria outlined in the Helsinki Declaration [22].

### 2.2. The Selection of Subjects Included in This Study

The selection of study participants was based on a set of inclusion/exclusion criteria. The inclusion criteria for adult subjects were as follows:

1. age equal to or over 50 years old;
2. confirmed cardiovascular pathology;
3. at least one missing tooth;
4. subjects who have never worn dentures;
5. subjects without one or more dental implants inserted.

The exclusion criteria were as follows:

1. age under 50 years of age;
2. subjects without confirmed cardiovascular pathology;
3. full dental arches or subjects with dentures;
4. subjects with one or more dental implants inserted [23].

Each subject selected to be part of this study was informed regarding the objectives of the present study and expressed their consent in writing.

### 2.3. Dental Examination

The data regarding oral status were obtained through a clinical examination, carried out by a trained dentist, respecting the WHO standards [24] and the Helsinki Declaration [22].

Each participant was examined while seated on the dental chair in upright position, with the head maintained in a normal position, using both natural light and light emitted by the dental unit lamp, as well as a mirror (3 12 *p*./20 mm Carl Martin GmbH, Solinger Germany) and a dental probe (4356 Kohler Medizintechnik, Stockach, Germany). The maximum number of teeth was 28, and the presence or absence of third molars, impacted teeth and residual deciduous teeth, were not considered [6]. All subjects included in this study were characterized by the presence of at least one tooth.

### 2.4. Data Collection

The study participants’ data, collected from the observation records belonging to the cardiology department, included the following: age, gender, place of origin, type of cardiovascular disease and presence of other systemic conditions. Based on these observations, the following general variables and associated secondary groups were defined:

• age group, associated with 4 secondary groups: 50–59 years old, 60–69 years old, 70–79 years old and over 80 years;
• gender, defining two secondary groups: males and females;
• residence, associated with two secondary lots: urban and rural;
• the type of cardiovascular disease, associated with 5 secondary groups: arterial hypertension (AHT), heart valve diseases, cardiomyopathies, coronary heart diseases and cardiac arrhythmias;
• the presence of another systemic comorbidity, associated with 4 secondary groups: pulmonary disorders, metabolic disorders, chronic renal failure or anasarca, and others.

Following the dental examination, the oral parameter reflecting the NRT was recorded, according to which the study participants were divided into 2 groups:

• the control group, which included subjects characterized by the presence of at least 21 teeth on both arches [15,25], as dentition with at least 21 teeth is considered to be functional [26];
• the study group, which included subjects with less than 21 teeth on both arches. According to the WHO definition, people with less than 21 teeth in the oral cavity are considered to have “non-functional dentition”, while people with at least 21 teeth on both arches are considered to have “functional dentition” [27].

### 2.5. Statistical Analysis

For all subjects included in this study, primary data were recorded using Microsoft Excel 365 (San Francisco, USA). Statistical analysis was performed using a software application dedicated to statistical processing: IBM SPSS Statistics 20.0 (IBM Corp., New York, NY, USA). Categorical data were expressed as absolute and relative frequencies (%), and continuous variables were expressed as mean ± standard deviation (SD). Normality was assessed using the Kolmogorov–Smirnov/Shapiro–Wilk test for all continuous data series. To assess the independent significance of teeth presence, compared to the threshold of 21 present teeth, with the outcome represented by a cardiovascular disorder category, the association of each group was evaluated by univariate (unadjusted) and multiple (adjusted with demographic and clinical features) logistic regression (LR) analyses, considering the end outcome (no disease = 0 and disease = 1) as the dependent variable and the teeth group as the independent parameter. Two binomial regression models were developed to assess the effects of demographic data (Model 1—partial model) and demographics plus comorbidities (Model 2—complete model) on the likelihood that participants have a specific cardiovascular disease. Linearity of the continuous data with respect to the logit corresponding to the dependent variable was determined via the Box–Tidwell (1962) procedure. The Bonferroni correction was applied considering all terms in the models, thus resulting different statistical thresholds for both models: *p* < 0.01 for the first model, and *p* < 0.00714 for the second model. Based on this assessment, all continuous independent variables were linearly related to the logit corresponding to the dependent variable. The following *p* value was accepted: *p* < 0.05 significance based on a confidence interval (CI) of 95%.

## 3. Results

The descriptive statistical analysis of the group of 84 subjects (40 males and 44 females) included in this study showed that the participants were aged between 50 and 89 years (mean ± SD: 69.49 ± 10.09). The distribution of the study participants according to the defined general variables and associated secondary groups is shown in Table 1.

**Figure 1:**
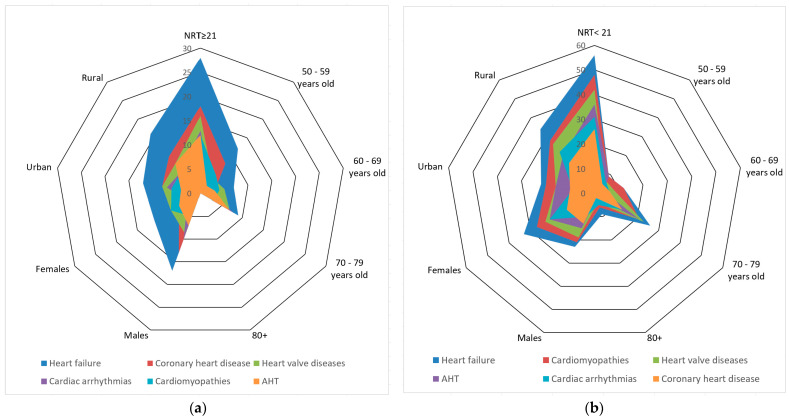
Distribution of participants according to NRT, general variables and degree of overlap of the number of cardiovascular diseases: (**a**) control group with subjects with NRT ≥ 21; (**b**) study group with subjects with NRT < 21.

The NRT on both arches ranged from 2 to 25 (mean 15.54 ± SD = 5.43). According to the NRT, the distribution of subjects was as follows: 33.33% (*n* = 28) of participants were included in the control group with an NRT ≥ 21, and 66.67% (*n* = 56) of them were included in the study group with an NRT < 21. The data collected from the cardiological observation sheets indicated that all the subjects participating in this study were diagnosed with heart failure. Therefore, each participant presented with at least two cardiovascular diseases. The distribution of participants according to the number of teeth, general variables and degree of overlap in the number of cardiovascular diseases is presented in Figure 1.

Next, the statistical analyses were carried out for the categories of subjects with cardiovascular diseases mentioned when defining the variables.

Taking into account the NRT, the comparative distribution of subjects in the control and study groups according to the presence or absence of a type of CVD is shown in Table 2. The control group is considered as a reference point.

The unadjusted oral parameter of an NRT < 21 may represent a statistically significant predictor for the development of cardiomyopathy (OR = 8.00, 95%CI = 2.78–23.06, *p* < 0.0005). This is also valid when considered in association with the general variables that were studied (Table 3 and Table 4). A value of NRT < 21 may also be considered as a predictor of heart valve diseases and arterial hypertension, in association with other comorbidities (except for metabolic or pulmonary comorbidities) (Table 4). The regression analyses revealed a borderline-significant association between the NRT, adjusted with metabolic comorbidities, and coronary disease (OR = 0.37, 95%CI = 0.13–1.01, *p* = 0.052).

### 3.1. Analysis of Model 1—Partial Model

For AHT, heart valve diseases, coronary diseases and cardiac arrhythmias, the LR model was not statistically significant (*p* > 0.05). The four predictor variables representing age, gender, residence and NRT were not statistically significant.

In the case of cardiomyopathy, three standardized residuals with values close to 2.5 standard deviations were identified, and they were kept within the analysis. This partial LR model was statistically significant (χ^2^(4) = 21.445, *p* < 0.0005). The LR model explained 32.3% (Nagelkerke) of the variance seen for cardiomyopathies; it also correctly classified 75% of cases. The predictor variables of age, gender and residence were not statistically significant. Only the NRT was statistically significant, as subjects with an NRT < 21 had 15.56 times higher odds of exhibiting cardiomyopathy, compared to subjects with an NRT ≥ 21.

### 3.2. Analysis of Model 2—Complete Model

For coronary diseases and cardiac arrhythmias, the LR model was not considered statistically significant (*p* > 0.05). Also, no predictor variable (NRT, demographic or clinical data) was statistically significant.

For heart valve diseases, the LR model was statistically significant (χ^2^(7) = 23.725, *p* = 0.003). This model explained 34.7% (Nagelkerke) of the variance seen in heart valve diseases and it correctly classified 72.6% of all cases. Of the nine predictor variables, only three of them were statistically significant: the presence of less than 21 teeth, pulmonary comorbidities and other comorbidities. Subjects with an NRT < 21 had 10.29 times higher odds of exhibiting heart valve diseases, compared to subjects with an NRT ≥ 21, while subjects with pulmonary comorbidities and other comorbidities had around 5 times higher odds of developing heart valve diseases, compared to subjects without them.

For cardiomyopathy, three standardized residuals with values close to 2.5 standard deviations were identified, and they were kept within the analysis. The LR model was statistically significant (χ^2^(7) = 45.006, and *p* < 0.0005). The model explained 59.4% (Nagelkerke) of the variance seen within the cardiomyopathy category, and it correctly classified 84.5% of all cases. Of the nine predictor variables, three were statistically significant. From the entire study lot, subjects with an NRT < 21 had 78.23 times higher odds to present with cardiomyopathy compared to subjects with an NRT ≥ 21, while subjects with other comorbidities had 25.58 times higher odds of presenting with cardiomyopathy compared to subjects without them. Similarly, increasing age was associated with an increase of 1.163 in the likelihood of exhibiting cardiomyopathy.

For AHT, two standardized residuals with values close to 2.5 standard deviations were identified, and they were kept within the analysis. The final LR model was statistically significant (χ^2^(7) = 26.153, *p* = 0.001). This model explained 35.9% (Nagelkerke) of the variance seen in AHT, and it correctly classified 70.2% of cases. Of the nine predictor variables, only two were statistically significant. Subjects with an NRT < 21 had 4.440 times higher odds of presenting with AHT than subjects with an NRT ≥ 21, and subjects with other comorbidities had around 10 times higher odds of exhibiting AHT than subjects without them. The presence of metabolic comorbidities correlated with a reduction in the likelihood of presenting with AHT.

Overall, the NRT may be considered a predictive marker that is relative to the risk of exhibiting cardiovascular conditions (Table 5).

## 4. Discussion

Correlations between the NRT and CVDs are significant both for individuals requiring various dental treatments, and for dental practitioners. Informing the beneficiaries of dental treatments about these correlations may draw their attention to the importance of maintaining the teeth on the dental arches, through appropriate preventive and therapeutic measures. From the point of view of dentists, data about the correlations between the NRT and CVDs may draw their attention to the fact that subjects with a low NRT have an increased cardiovascular risk. That is why the dental management of subjects with a low NRT, and the types of treatments performed on them, must be appropriate. In addition, the risk of cardiovascular disorders must be considered during therapeutic dental procedures. In this regard, Findler et al. conducted a study on 54 subjects with heart failure at various stages who underwent emergency dental treatments to eliminate either pain or the source of an oral infection [28]. The authors specified that the dental management of these patients focused on the prevention of iatrogenic factors, induced by the context of the dental office, and precipitants of heart failure. The results of this study indicated that all planned dental procedures were completed successfully, but six patients presented with respiratory distress and five presented with arrhythmias during the dental treatment. Regarding blood pressure and heart rate, only minimal differences between the groups were revealed [28].

In this study, the distribution of subjects suffering from cardiovascular diseases, according to their NRT, indicated that 2 out of 3 (2/3) (66,67%) participants were included in the study group study for individuals with an NRT < 21 and, based on literature data, they were classified in the category of those suffering from non-functional dentition [27]. Toniazzo et al. have demonstrated that individuals with a low average number of teeth/functional dental units have a poor nutritional status, compared to subjects with a greater number of tooth pairs, who present with a good nutritional status [29]. Contrary to these results, the study conducted by Khoury et al. emphasized the fact that a decrease of one unit in the number of posterior occluding tooth pairs was associated with variations in nutritional status, as assessed by univariate statistical analysis. However, this association was no longer relevant after the statistical adjustment [30].

The study conducted by Shiga et al., with a focus on the relationship between tooth loss, nutritional status and the occurrence of stroke, demonstrated using a multivariate analysis that tooth loss correlated with poor nutritional status, and both were independently associated with poor functional outcomes after a severe/acute ischemic stroke [31]. Another synthesis and meta-analysis study further associated the Controlling Nutritional Status (CONUT) score and the occurrence of major adverse cardiovascular events in individuals suffering from coronary artery disease. The results highlight that undernourished subjects (defined by a CONUT score of ≥2) present with a 52% higher risk of major adverse cardiovascular events compared to the subjects with normal nutrition. The study concluded that estimating nutritional status through the CONUT score could be useful for improving the risk classification of coronary artery disease [32]. In this study, it was observed that for subjects with ”non-functional dentition“ (NRT < 21), the unadjusted dental parameter was not a predictive factor for coronary artery diseases, but the regression statistical tests indicated a significant association between these conditions and NRT < 21 that was adjusted for metabolic comorbidities. These results correspond with the literature data previously mentioned, with the subjects with “non-functional dentition” possessing a higher risk for the occurrence of coronary artery diseases compared to those with functional dentition.

Regarding the study participants with heart rhythm disorders, it was noticed that the oral parameter of NRT did not serve as a predictive marker, because the unadjusted statistical analysis or logistic regression did not reveal a correlation for the group studied. Studies in the literature have addressed the association between the number of missing teeth and heart rhythm disorders. Cheng et al. emphasized, through a univariate analysis, the dose–response relationship between an increasing number of missing teeth and an increasing risk of atrial fibrillation (AF) and heart failure (unadjusted HR; 95% CI) for AF. The multivariate analysis has only shown an association between an increasing number of missing teeth number and heart failure [33]. Leelapatana et al. suggest that further investigations are needed regarding the association between the occurrence of atrial fibrillation and other indicators of oral health, for example, dental caries and tooth loss, in order to clarify the underlying mechanisms of this association [34].

In the present study, for cardiac valve disorders, an NRT < 21 served as a predictive marker in the univariate analysis. This was also true for the subjects with other comorbidities, except for the metabolic and pulmonary ones. The complete model for the logistic regression analysis highlighted that subjects with an NRT < 21 had a 10.29 times higher probability of developing valvular heart diseases than those with an NRT ≥ 21. Additionally, in subjects with an NRT < 21 and the presence of pulmonary comorbidities or those categorized as ”others“, the risk of the occurrence of valvular heart diseases increased by 5 times compared with subjects with an NRT < 21, but without these comorbidities. These results are similar to those obtained by Terano et al. The authors analyzed, through LR, the association between the number of remaining teeth and the incidence of postoperative respiratory complications in patients who have undergone a cardiac valve surgery. The results showed that subjects with <20 remaining teeth had a significantly higher incidence of postoperative respiratory complications than those with ≥20 remaining teeth, with an odds ratio of 29.800 (*p* = 0.004) [35].

For subjects with cardiomyopathies, an NRT < 21 served as a predictive factor, both in the univariate statistical analysis, and through LR, in both the complete and partial models. Subjects with an NRT < 21 had a probability risk for developing cardiomyopathy that was 78.23 times higher than the subjects with an NRT ≥ 21. Among subjects with non-functional dentition, the presence of comorbidities and aging also increase the risk of disease occurrence. Conversely, the results of the study conducted by Ziebolz et al. did not confirm any association between the cardiomyopathy type and the number of remaining teeth, supporting the lack of systemic disease effects on periodontal health in individuals with severe heart conditions [36].

For subjects with AHT, an NRT < 21 served as a predictive factor in the univariate statistical analysis and through logistic regression in the complete model. Furthermore, the presence of comorbidities increased the risk of developing AHT, with the exception of metabolic comorbidities. The partial logistic regression analysis model did not reveal an association between the NRT and the risk of developing AHT. Similarly to the present study, in the research conducted by Shin, the limit of <20 of remaining teeth was used to define severe tooth loss. In this context, the relationship between the remaining teeth number and the diastolic blood pressure followed a nonlinear pattern, increasing in individuals with 20 to 27 remaining teeth, and decreasing in those with less than 19 teeth. The author concluded that a decreasing number of remaining teeth may be independently associated with arterial hypertension in the Korean population [37]. Peres et al. demonstrated that, in individuals with less than 10 remaining teeth, the number of teeth was associated with an increased systolic blood pressure [38]. Del Bruto et al. used the same limit of 10 remaining teeth to define severe tooth loss. Moreover, they demonstrated the presence of a direct association between severe tooth loss and the systolic component of blood pressure [39]. The possible mechanisms responsible for the apparent lack of association between severe tooth loss and diastolic blood pressure are still unclear.

The strength of this article is represented by the fact that the NRT evaluation was performed by a trained investigator, and not by the study participants through self-reporting. Also, the study participants were diagnosed with CVDs by a cardiologist during their hospitalization.

The limitation of this study is represented by the number of subjects included in this study, who were all hospitalized in the cardiology department, and who presented themselves in the dental emergency service within the same medical unit. This fact can have a potential impact on the results. Different results may have been obtained if this research was carried out on a larger number of hospitalized subjects who were selected from several hospitals. Also, in hospitalized subjects, the NRT parameter may present a different variability pattern compared to non-hospitalized subjects, which may influence the association of NRT with a certain type of CVD.

Future research directions will focus on the evaluation of cardiovascular disorders occurring during dental treatments in subjects diagnosed with CVDs, and on the expansion of the sample studied.

## 5. Conclusions

This study argues for the importance of a simple and rapid assessment of the dental parameter of NRT, with a role in guiding dentists on the cardiovascular risk of individuals requiring emergency dental treatments.

A low number of remaining teeth can be considered to be a relative predictive marker for the risk of exhibiting cardiovascular conditions, especially heart valve diseases, cardiomyopathy and hypertension. The gender of the subjects and their type of residence did not influence the correlation between the number of teeth and CVDs, but an increasing age determined the increase in the risk of exhibiting cardiomyopathy in subjects with a low number of remaining teeth. The presence of pulmonary comorbidities influenced the association between the number of remaining teeth and heart valve disease, and metabolic comorbidities influenced the association between the number of remaining teeth and hypertension.

## Figures and Tables

**Table 1:** The distribution of the study participants according to the defined general variables.

No	General Variable								
1	Age group								
	Secondary groups	50–59 years		60–69 years		70–79 years		Above 80 years	
	No. (%)	21 (25.00%)		19 (22.62%)		35 (41.67%)		9 (10.71%)	
2	Gender								
	Secondary groups	Male				Female			
	No. (%)	40 (47.62%)				44 (52.38%)			
3	Residence area								
	Secondary groups	Urban areas				Rural areas			
	No. (%)	34 (40.48%)				50 (52.59%)			
4	Type of CVD								
	Secondary groups	Valve diseases	Coronary heart disease		Rhythm disorders		Cardiomyopathies		Hypertension
	No. (%)	58 (59.05%)	44 (52.38%)		44 (52.38%)		60 (71.43%)		48 (57.14%)
5	Other associated systemic comorbidities								
	Secondary groups	Metabolic	Pulmonary		Chronic renal failure		Anasarca		Other disease
	No. (%)	36 (42.86%)	64 (76.19%)		12 (14.29%)		18 (21.43%)		22 (26.19%)

**Table 2:** Distribution of subjects according to NRT and general variables.

Parameter	Category	Control Group with Subjects with NRT ≥ 21	Study Group with Subjects with NRT< 21	TOTAL
Age	50–59	12 (57.1%)	9 (42.9%)	21 (100%)
	60–69	7 (36.8%)	12 (63.2%)	19 (100%)
	70–79	9 (25.7%)	26 (74.3%)	35 (100%)
	80+	0 (0.0%)	9 (100%)	9 (100%)
Gender	Male	17 (42.5%)	23 (57.7%)	40 (100%)
	Female	11 (25.0%)	33 (75.0%)	44 (100%)
Residence	Rural	16 (32.0%)	34 (68.0%)	50 (100%)
	Urban	12 (35.3%)	22 (64.7%)	34 (100%)
Type of cardiovascular disease	Heart valve diseases	16 (27.6%)	42 (72.4%)	58 (100%)
	Coronary heart diseases	18 (40.9%)	26 (59.1%)	44 (100%)
	Cardiomyopathies	12 (20.0%)	48 (80.0%)	60 (100%)
	Cardiac arrhythmias	13 (29.5%)	31 (70.5%)	44 (100%)
	AHT	12 (25.0%)	36 (75.0%)	48 (100%)
Comorbidities	Metabolic comorbidities	9 (25.0%)	27 (75.0%)	36 (100%)
	Pulmonary comorbidities	20 (31.3%)	44 (68.8%)	64 (100%)
	Renal insufficiency and anasarca	8 (28.6%)	20 (71.4%)	28 (100%)
	Other comorbidities	4 (18.2%)	18 (81.8%)	22 (100%)

**Table 3:** Unadjusted and adjusted regression analyses on associations between NRT, gender, age, residence and type of CVD.

Cardiac Condition	Study Group (NRT < 21) OR (95% CI)							
	No Adjustment		Gender		Age		Residence	
	OR (95% CI)	*p*	OR (95% CI)	*p*	OR (95% CI)	*p*	OR (95% CI)	*p*
Valve heart disease	2.25 (0.86–5.89)	0.099	2.37 (0.88–6.36)	0.087	1.99 (0.69–5.77)	0.204	2.26 (0.86–5.94)	0.097
Coronary heart disease	0.48 (0.19–1.27)	0.125	0.56 (0.21–1.46)	0.235	0.54 (0.19–1.52)	0.245	0.48 (0.19–1.23)	0.126
Cardio- myopathy	8.00 (2.78–23.06)	<0.0005	7.61 (2.61–22.20)	<0.0005	15.08 (3.81–59.62)	<0.0005	8.50 (2.88–25.08)	<0.0005
Cardiac arrhythmia	1.43 (0.58–3.56)	0.441	1.39 (0.55–3.53)	0.480	1.14 (0.51–3.80)	0.521	1.48 (0.57–3.53)	0.455
AHT	2.40 (0.95–6.06)	0.064	2.39 (0.93–6.15)	0.070	2.10 (0.76–5.82)	0.152	2.41 (0.95–6.11)	0.063

**Table 4:** Unadjusted and adjusted regression analyses on associations between NRT, systemic comorbidities and type of CVD.

Cardiac Condition	Study Group (NRT < 21) OR (95% CI)							
	Renal Insufficiency and Anasarca		Metabolic Comorbidities		Pulmonary Comorbidities		Other Comorbidities	
	OR (95% CI)	*p*	OR (95% CI)	*p*	OR (95% CI)	*p*	OR (95% CI)	*p*
Valve heart disease	2.23 (0.85–5.86)	0.103	2.67 (0.97–7.38)	0.060	2.43 (0.91–6.50)	0.080	3.75 (1.22–11.48)	0.020
Coronary heart disease	0.50 (0.19–1.30)	0.155	0.37 (0.13–1.01)	0.052	0.45 (0.17–1.16)	0.100	0.46 (0.18–1.19)	0.110
Cardio- myopathy	8.36 (2.78–25.12)	<0.0005	8.50 (2.86–25.29)	<0.0005	8.58 (2.80–26.31)	<0.0005	12.11 (3.51–41.8)	<0.0005
Cardiac arrhythmia	1.35 (0.52–3.46)	0.536	1.96 (0.72–5.32)	0.190	1.38 (0.55–3.46)	0.500	1.56 (0.61–3.98)	0.350
AHT	2.41 (0.95–6.11)	0.063	2.22 (0.88–5.69)	0.100	2.32 (0.90–5.96)	0.080	4.25 (1.46–12.43)	0.010

**Table 5:** Adjusted regression on associations between NRT, general variables and CVD.

Cardiac Condition	NRT Adjusted with General Variables (Partial Model)		NRT Adjusted with General Variables (Complete Model)	
	OR (95% CI)	*p*	OR (95% CI)	*p*
Valve heart disease	2.09 (0.71–6.19)	0.181	10.29 (2.110–50.219)	0.004
Coronary heart disease	0.60 (0.21–1.74)	0.350	78.23 (10.280–390.254)	<0.0005
Cardiomyopathy	15.56 (3.82–63.34)	<0.0005	0.27 (0.070–1.035)	0.056
Cardiac arrhythmias	1.34 (0.49–3.72)	0.569	2.913 (0.793–10.696)	0.107
AHT	2.12 (0.76–5.95)	151	4.440 (1.183–16.665)	0.027

## Data Availability

The authors declare that the data associated with this research are available from the corresponding authors upon reasonable request.
